# Effects of aerobic exercise therapy and cognitive behavioural therapy on functioning and quality of life in amyotrophic lateral sclerosis: protocol of the FACTS-2-ALS trial

**DOI:** 10.1186/1471-2377-11-70

**Published:** 2011-06-14

**Authors:** Annerieke C van Groenestijn, Ingrid GL van de Port, Carin D Schröder, Marcel WM Post, Hepke F Grupstra, Esther T Kruitwagen, Harmen van der Linde, Reinout O van Vliet, Margreet GH van de Weerd, Leonard H van den Berg, Eline Lindeman

**Affiliations:** 1 Rudolf Magnus Institute of Neuroscience and Centre of Excellence for Rehabilitation Medicine, University Medical Centre Utrecht and Rehabilitation Centre De Hoogstraat, the Netherlands; 2 Swiss Paraplegic Research, Nottwil, Switzerland; 3 Het Roessingh Rehabilitation Centre, Enschede, The Netherlands; 4 Department of Rehabilitation Medicine, Academic Medical Centre, Amsterdam, the Netherlands; 5 Radboud University Medical Centre, Nijmegen, the Netherlands

## Abstract

**Background:**

Amyotrophic lateral sclerosis (ALS) is a fatal progressive neurodegenerative disorder affecting motor neurons in the spinal cord, brainstem and motor cortex, leading to muscle weakness. Muscle weakness may result in the avoidance of physical activity, which exacerbates disuse weakness and cardiovascular deconditioning. The impact of the grave prognosis may result in depressive symptoms and hopelessness. Since there is no cure for ALS, optimal treatment is based on symptom management and preservation of quality of life (QoL), provided in a multidisciplinary setting. Two distinctly different therapeutic interventions may be effective to improve or preserve daily functioning and QoL at the highest achievable level: aerobic exercise therapy (AET) to maintain or enhance functional capacity and cognitive behavioural therapy (CBT) to improve coping style and cognitions in patients with ALS. However, evidence to support either approach is still insufficient, and the underlying mechanisms of the approaches remain poorly understood. The primary aim of the FACTS-2-ALS trial is to study the effects of AET and CBT, in addition to usual care, compared to usual care alone, on functioning and QoL in patients with ALS.

**Methods / Design:**

A multicentre, single-blinded, randomized controlled trial with a postponed information model will be conducted. A sample of 120 patients with ALS (1 month post diagnosis) will be recruited from 3 university hospitals and 1 rehabilitation centre. Patients will be randomized to one of three groups i.e. (1) AET + usual care, (2) CBT + usual care, (3) Usual care. AET consists of a 16-week aerobic exercise programme, on 3 days a week. CBT consists of individual psychological support of patients in 5 to 10 sessions over a 16-week period. QoL, functioning and secondary outcome measures will be assessed at baseline, immediately post intervention and at 3- and 6-months follow-up.

**Discussion:**

The FACTS-2-ALS study is the first theory-based randomized controlled trial to evaluate the effects, and the maintenance of effects, of AET and CBT on functioning and QoL in patients with ALS. The results of this study are expected to generate new evidence for the effect of multidisciplinary care of persons with ALS.

**Trial registration:**

Dutch Trial Register NTR1616.

## Background

Amyotrophic lateral sclerosis (ALS) is a fatal progressive neurodegenerative disorder affecting motor neurons in the spinal cord, brainstem, and motor cortex. Patients are afflicted by progressive wasting and weakness of limb, bulbar, and respiratory muscles, and die on average within 3 years after symptom onset, usually because of respiratory failure [1]. In the absence of an established biological marker, the diagnosis of ALS is primarily clinical, based on El Escorial criteria [2]. The median age of onset of ALS is 55 years [1]. The incidence of ALS is between 1.5 and 2.5 per 100,000 person-years of follow-up in industrialized countries [3], and life-time risk of ALS is estimated to be between 1/600 and 1/2000 [1,4], which makes it the most common motor neuron disease.

ALS is familial in 5% of cases, with a Mendelian pattern of inheritance. The clinical phenotype of familial ALS (FALS) is similar to that of the sporadic form of the disease. At least 13 genes of major effect have been associated with FALS, accounting for 30% of familial cases. Sporadic ALS is considered to be a complex genetic disease, in which genetic and environmental factors combine to increase the disease risk [5].

Symptoms presented in the early stages of ALS may vary, and typically result from the combination of lower motor neuron loss (atrophy, fasciculations, weakness) and upper neuron motor loss (spasticity, pathological reflexes) [1,6]. Most patients show either progressive asymmetric focal weakness of the upper and lower extremities, e.g. poor handgrip or stumbling (80%), or bulbar symptoms, e.g. dysarthria, dysphagia (20%) [1,6]. Muscle weakness caused by ALS may also result from the avoidance of physical activity, which may result in cardiovascular deconditioning and disuse weakness, superimposed on the weakness caused by the ALS itself [7]. If the reduced level of activity persists, further deconditioning can develop, and muscle and joint tightness may lead to contractures and pain. All these aspects hamper daily activities [7]. Besides physical limitations, cognitive function deficits in a frontotemporal pattern may be apparent, including impaired frontal executive abilities in up to 50% of patients [8]. The grave prognosis of ALS can have a severe psychological impact on patients and their social environment. The majority of patients report not only physical but also existential problems [9]. Depressive symptoms [10] and hopelessness [10,11] are more common in patients with ALS than in the general population.

To date, no curative treatment is available. Because ALS symptoms are multifactorial, usual care for patients with ALS in the Netherlands consists of multidisciplinary care by specialized ALS care teams, coordinated by a rehabilitation physician.

The aim of our study is to improve or preserve functioning and quality of life (QoL) in patients with ALS. "Functioning" in this study refers to the domains of "activities" and "participation", as defined in the International Classification of Functioning, Disability and Health (ICF) [12], including functional status, physical activity, participation restrictions, and autonomy in participation. "QoL" is a construct described as "an individual integration of physical aspects such as symptoms of illness or wellness, psychological aspects such as emotional responses and beliefs, and social aspects such as interpersonal relationships and social support" [13]. Two distinctly different therapeutic interventions may be hypothesized to support the QoL of patients with ALS and preserve functioning at the highest achievable level, viz. aerobic exercise therapy (AET) to maintain or increase functional capacity, and cognitive behavioural therapy (CBT) to improve coping style and cognitions in patients with ALS. However, evidence to support the effectiveness of either approach is still limited.

The practice of prescribing exercise programmes for people with ALS is controversial, as some experts have discouraged exercise programmes for patients with neuromuscular disease for fear of "overuse weakness" [14-16]. Epidemiologic data showing a higher incidence of ALS in people who had engaged in intense physical activity during work or leisure before the onset of the disease has led to reluctance to prescribe exercise for people with ALS [17].

On the other hand, in a recent Cochrane review on muscle strength training and aerobic exercise training for patients with neuromuscular diseases (NMD), Voet [18] concluded that moderate-intensity strength training appeared not to be harmful to patients with myotonic dystrophy (MD) and facioscapulohumeral muscular dystrophy (FSHD), though there was insufficient evidence to establish its benefits. In a systematic review, Cup [19] summarized and critically appraised the available evidence on exercise therapy and other types of physical therapy for patients with NMD, including ALS. Again, exercise training did not seem to harm these patients, but insufficient evidence was reported for the effects of strengthening exercises, aerobic exercises or lifestyle modifications in the domains of body functions, activities, or participation for patients with ALS. The Cochrane review by Dal Bello [7] included only two small RCTs on exercise training for patients with ALS, and the small sample sizes made it impossible to draw conclusions about the effect of strengthening exercises for people with ALS. The authors recommended conducting larger, controlled studies to elucidate the effect of strengthening or aerobic exercise in people with ALS. In addition, better controlled studies are needed to determine which exercise protocols are most beneficial or cause undue risks, and whether there is a sub-set of people with ALS who respond more favourably to exercise, both physically and psychologically [7].

In addition to AET, it might be beneficial to alter coping styles and illness cognitions in patients with ALS by means of cognitive behavioural therapy (CBT), in order to improve or preserve functioning and QoL. Coping style and illness cognitions seem to be much more important than disease severity for the QoL of patients with ALS [20-22]. A passive coping style is a strong and consistent determinant of decreased QoL in patients with ALS and their primary caregivers [22]. In a cross-sectional study by Matuz [21], multiple regression analysis revealed that 56% of the variance of QoL was explained by social support, coping strategies, and cognitive appraisal. Another psychological factor that has been shown to explain variance in activity limitations in patients with ALS is emotional wellbeing [23]. A psychological intervention like CBT, aiming to optimize coping style and cognitions, has been proven to be effective in terms of QoL in patients with multiple sclerosis suffering from depression [24], and in terms of physical functioning in patients with chronic fatigue syndrome [25,26] and severely fatigued disease-free cancer patients [27]. This may suggest that CBT can also be beneficial to patients with ALS. However, no evidence for the effectiveness of CBT in patients with ALS is currently available.

The primary objectives of the FACTS-2-ALS trial are:

- Studying the effects of AET in addition to usual care, compared to usual care alone, on functioning and QoL in patients with ALS.

- Studying the effects of CBT in addition to usual care, compared to usual care alone, on functioning and QoL in patients with ALS.

Secondary objectives of the FACTS-2-ALS trial are:

- Studying the effects of AET in addition to usual care in patients with ALS on the ICF domains of body functions, and environmental and personal factors.

- Studying the effects of CBT in addition to usual care in patients with ALS on the ICF domains of body functions, and environmental and personal factors.

## Methods/Design

### Study design

**Figure 1:**
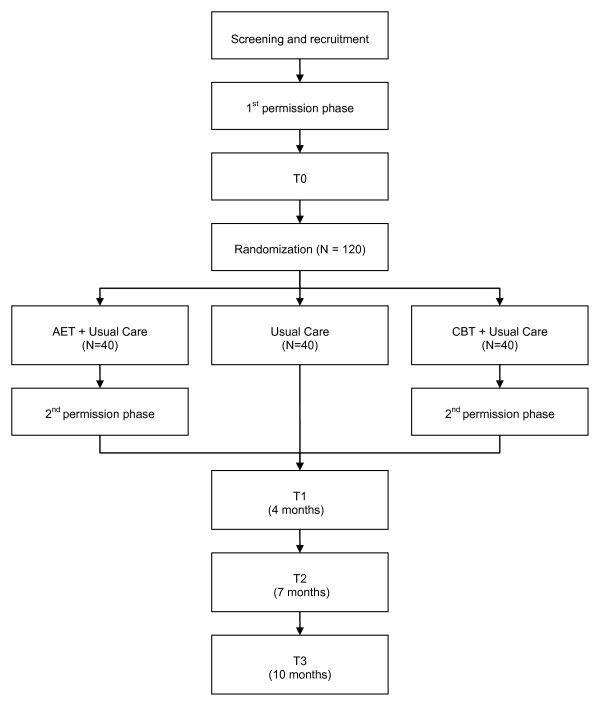
**Study design**.

A multicentre, single-blinded, randomized controlled trial (RCT) with 6 months follow-up will be conducted to evaluate the effects of aerobic exercise therapy (AET) in addition to usual care, or cognitive behavioural therapy (CBT) in addition to usual care, compared to usual care alone, in patients with ALS (figure 1). Participants will first be invited to participate in a longitudinal study including four repeated measurements (first permission phase): at entry (T0), at 4 months (T1), at 7 months (T2), and at 10 months (T3). After randomization to one of the intervention groups, the participants will be informed about the intervention they have been assigned to, and will be asked to participate in this intervention (second permission phase). The interventions will take place between T0 and T1, making T2 a short-term and T3 a long-term follow-up for those who participate in an intervention. The patients in the usual care group will not be asked additional permission. This postponed information model [28] allows us to inform participants about part of the study only, while the rest of the information will be given to them at the end of the study.

The study protocol has been approved by the Medical Ethics Committee of the University Medical Centre Utrecht (UMCU), and all participating centres have agreed to participate. Informed consent according to the declaration of Helsinki will be obtained from all participants (in both the first and second permission phases).

The FACTS-2-ALS trial is part of the FACTS-2-NMD project, along with the FACTS-2-PPS [29] and FACTS-2-FSHD [30] trials. An additional study will analyse qualitative data on patients' and partners' expectations of, and experiences with, the interventions, using a responsive methodology for all 3 trials [31].

### Study population

The aim is to include 120 patients with ALS, one month post diagnosis, who meet the inclusion and exclusion criteria (table 1). All patients will be recruited from outpatient clinics of the UMCU, the Academic Medical Centre (AMC) in Amsterdam, the University Medical Centre Nijmegen (UMCN) or het Roessingh Rehabilitation Centre in Enschede. The neurologists will refer eligible patients to a rehabilitation physician, who will check the inclusion and exclusion criteria. If a patient meets all the criteria, the rehabilitation physician will inform them about the four measurements, and will ask permission for them to be contacted by the primary investigator. The primary investigator will invite the patients by telephone to take part in the four measurements (first permission phase), and (for those patients randomized to one of the interventions) to participate in the relevant intervention (second permission phase).

**Table 1:** Inclusion and exclusion criteria

Inclusion criteria
(1) age between 18 and 70 years

(2) life-expectancy longer than one year

(3) forced vital capacity at least 80%

(4) diagnosis of "Probable" of "definite" ALS according to the "revised El Escorial WFN criteria" [2]

(5) at least 1 month after the diagnosis of ALS

(6) being in the rehabilitation phase; diagnostic phase is completed

(7) walking ability with or without a ankle-foot orthosis or stick, and cycling ability on a cycle ergometer, so that the intervention can be expected to be completed

**Exclusion criteria**

(1) cognitive impairment

(2) insufficient mastery of the Dutch language

(3) disabling co-morbidity interfering with the intervention programmes or influencing outcome parameters (including severe cardiopulmonary disease, like chest pain, arrhythmia, pacemaker, cardiac surgery, severe dyspnoea on effort or emphysema, epileptic seizures, poorly regulated diabetes mellitus or hypertension

(4) psychological disorder preventing the intervention from being completed

### Randomization and blinding

Eligible participants will be randomized to one of three groups: (1) AET + usual care, (2) CBT + usual care, or (3) usual care. Patients who exercise for two or more hours a week will be excluded from the AET intervention, due to the small expected treatment effect, but can be randomized to the CBT or control group. In addition, since CBT is not expected to be necessary for patients who show no anxious or depressive symptoms, patients who score less than 8 on the Hospital Anxiety and Depression Scale (HADS) [32] will be excluded from the CBT intervention, but can be randomized to the AET or control group. All participating patients will be registered and randomized by one independent person. Randomization of the three trial arms will be implemented by the "minimization" method [33], a dynamic randomization algorithm that ensures even distribution of patients across strata, even if there are relatively small groups of patients to be divided over multiple strata. In this study, patients will be stratified by (a) study site (Amsterdam, Nijmegen, Enschede, Utrecht); (b) age; (c) the use of lithium; (d) type of ALS (spinal / bulbar); and (e) gender. A biphasic randomization of patients will be used, based on a postponed information model [28]. All outcomes will be assessed by blinded and independent research assistants. At the beginning of each assessment, patients will be instructed not to reveal their intervention to the blinded research assistant.

### Interventions

#### Usual Care

The patients in the control and intervention groups will receive usual care. In the Netherlands, patients with ALS receive multidisciplinary care from specialized ALS care teams consisting of a rehabilitation physician, a physical therapist, an occupational therapist, a speech pathologist, a dietician, and a social worker. The ALS team works according to the Dutch protocol for rehabilitative management in ALS [34]. Rehabilitation medicine plays an important role in the symptomatic and palliative treatment of patients with ALS. The purpose of the rehabilitation treatment is to reduce the impact of the disabilities, so that patients can fulfil the roles they may wish and need to. Maintaining QoL is the primary objective. Rehabilitation treatment includes instructions on safety in mobility, use of aids and appliances, orthoses, and psychosocial guidance. Patients with ALS often receive physical therapy periodically, whenever new mobility problems arise. This therapy usually comprises compensatory strategies. In later stages of the disease, patients mostly receive physical therapy at home by a physical therapist, including passive mobility exercises, with practical goals to help them function safely at home. If necessary, the rehabilitation physician refers a patient to a social worker for social support. If there is a particular presenting complaint regarding mood disturbances, the patient can be referred to a psychologist.

Patients included in our trial will not be restricted in terms of their treatment options in the context of usual care; co-interventions such as exercise therapy and/or psychotherapy will be monitored throughout the study by means of diaries.

#### Aerobic Exercise Therapy (AET)

The AET will consist of a 16-week aerobic exercise programme, on 3 days a week, twice a week at home (1) and once a week in an individually guided group session at a hospital (2). The therapy will be supervised by specially trained physiotherapists.

(1) The training programme at home will consist of individually tailored aerobic exercises on a cycle ergometer and a stepboard. Patients will receive a Polar RS400 heart rate watch with chest strap, a Kettler X7 cycle ergometer, a Kettler stepboard, a log book with training instructions and a training schedule to be used at home for the duration of the intervention. During each training session, their heart rate will be recorded continuously by a (Polar RS400) heart rate monitor. Furthermore, the patients will use the log book to document the number and duration of training sessions, the training intensity, perceived exertion on the Borg Rated Perceived Exertion (RPE) scale [35], and possible complaints experienced after the training session. Each session will consist of a cycle ergometer training, followed by a stepboard training. The duration of the cycle ergometer training sessions will be gradually increased from 15 to 30 minutes per session in the first four weeks, after which the duration of training will be maintained at 30 minutes. The duration of the stepboard training session will be gradually increased from 3 minutes (weeks 1 - 5) to 4 minutes (weeks 6 - 10) to 5 minutes (weeks 11 - 16) per session. This training structure is based on expert opinion, since there have been no previous studies on aerobic exercise training in persons with ALS. Training intensity will be gradually increased from 50% of the Heart Rate Reserve (HRR) to 75% HRR, in accordance with the American College of Sports Medicine guidelines [36] for aerobic training in healthy adults and persons with chronic diseases and disabilities [37]. HRR is the difference between the predicted maximum heart rate (220 - age) and the measured resting heart rate. The HRR is equivalent to the difference between maximal and resting maximal oxygen consumption (VO2max). Each participant will be instructed how to adjust the physical intensity to their prescribed individual heart rate. Warming-up and cooling-down will consist of 5 minutes unloaded cycling.

(2) The supervised group training sessions at the hospital will consist of workstations, including individually tailored aerobic exercises and muscle strengthening exercises. Sessions will be divided into a 5-minute warming-up period, 30 minutes of aerobic exercises (cycle ergometer, stepboard, and treadmill) and 20 minutes of muscle strengthening exercises (quadriceps, biceps, and triceps) and a 5-minute cooling-down period.

Aerobic exercise workstations: training intensity will be gradually increased from 50% of the HRR to 75% HRR. Exercise duration per workstation will remain constant, i.e. cycle ergometer 15 minutes; treadmill 10 minutes; stepboard 3 to 5 minutes. After each aerobic workstation, duration and heart rate (as determined by the heart rate monitor) as well as the perceived exertion on the Borg RPE scale, will be recorded in the log book by the physical therapist.

Muscle strengthening exercises at workstations: the maximum strength (1-RM) of different muscle groups in arms and legs will be determined individually using a submaximal test. Since muscle strength is expected to deteriorate over time in patients with ALS, the physical therapist will determine the maximum strength before the start of the programme, and again after 5 and 10 weeks of training, to ensure that the patients exercise at the correct training intensity. In the first 5 weeks, training intensity will be 40% of 1-RM, with each exercise session consisting of 3 series of 10 repetitions each, with 1 minute of rest between series. Training intensity will be increased to 50% of 1-RM in week 6, and in week 11 the number of repetitions will be increased to 15. After each muscle strengthening exercise, the therapist will record the actual training intensity achieved and the number of series and repetitions in the logbook, as well as the perceived exertion on the Borg RPE scale. This progressive, moderate intensity is in accordance with the intensity of the exercise programme for persons with ALS developed by Dal Bello [38].

Training duration and intensity of both the home-based training programme and the supervised group training schedules will be determined weekly and checked by the therapist by reading out the heart rate monitors, assessing the Borg RPE scale scores and checking the log books. If necessary, individually tailored adjustments to the training schedules will be made by the therapist.

#### Cognitive Behavioural Therapy (CBT)

**Figure 2:**
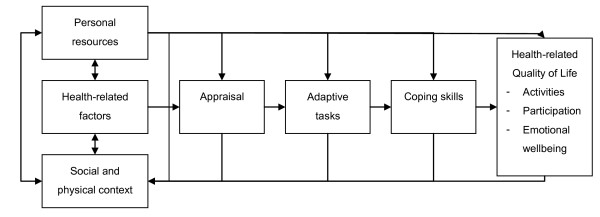
**Stress-coping model**.

Assuming that living with ALS constitutes stress, we based our CBT approach on a stress-coping model originally proposed by Lazarus & Folkman (figure 2) [39]. The impact of the symptoms of ALS and the lack of a curative treatment require adaptive mechanisms; patients have to adjust their internal needs to new external demands. The cognitive process by which this event is interpreted and evaluated is appraisal. Appraisal leads to adaptive tasks, which require coping skills. The required coping skills include a wide range of strategies aimed at stress reduction. Coping influences health-related outcomes such as activities and participation, and improves QoL.

The CBT intervention in the FACTS-2-ALS trial will aim to reduce stress by training effective coping skills, modifying dysfunctional cognitions, providing the right amount of disease- and support-related information and encouraging patients to seek social support. Therapy will be tailored to each individual patient and their partner, using behavioural and rational-emotive principles and comprising six "modules". These modules have been designed on the basis of interviews with ALS patients and caregivers, and include the six most important areas identified by the patients with ALS and caregivers who were interviewed: (1) coming to terms with the diagnosis of ALS; (2) coping with mood disorders; (3) maintaining autonomy; (4) mobilizing social support; (5) fear of the future; and (6) maintaining activity levels. Modules will be chosen in the first intake session by the therapists, based on patients' and partners' perceived problems. Depending on the problems, sessions will be held individually or together with the partners. Cognitive Behavioural Therapy (CBT) will consist of individual support for patient and partner by trained psychologists with experience in rehabilitation medicine. Depending on the perceived problems, the psychologists will determine, in consultation with the patients, the number of sessions (5 to 10) to be held during the 16-week period, each with a duration of 1 hour.

### Compliance and attrition

Compliance will be assessed by recording the number of treatment sessions (AET or CBT) attended. For the patients randomized to AET, the total time spent on aerobic exercise on the cycle ergometer and stepboard at home will also be recorded in a log book. Where applicable, participants will be asked to express their motivation for poor compliance or drop-out.

### Outcomes

Outcome measurements (table 2) will be obtained at entry into the study (T0), immediately after the 4-month intervention period (T1), at 3-month follow-up (short-term follow-up; T2), and at 6-month follow-up (long-term follow-up; T3). The primary outcome measure will be QoL, assessed with the ALS Assessment Questionnaire (ALSAQ-40) [40] and Short Form 36 (SF-36) [41], and functioning as assessed with the LASA Physical Activity Questionnaire (LAPAQ) [42], the Impact on Participation and Autonomy questionnaire (IPA) [43], and the Sickness Impact Profile 68 (SIP 68) [44]. Secondary outcome measures will be categorized in accordance with the International Classification of Functioning (ICF) [12] for the domains of body functions and environmental and personal factors. Outcome measures for the domain of body functions (disease severity, survival, cardio-respiratory fitness, functional capacity, muscle strength, lung function, fatigue, sleep disturbances, pain, blood pressure, and weight/height) and will be determined at the three University Medical Centres by a trained research assistant blinded for treatment allocation.

**Table 2:** Outcome measures and instrumentation

Primary outcome measures	Instrumentation	T0	T1	T2	T3
Quality of life	ALS Assessment Questionnaire (ALSAQ-40) [40]	X	X	X	X
	Short Form 36 (SF-36) [41]	X	X	X	X

***ICF: Activities / participation***					

Self-reported physical activity	LASA Physical Activity Questionnaire (LAPAQ) [42]	X	X	X	X

Limitations in participation and autonomy	The Impact on Participation and Autonomy questionnaire (IPA) [48]	X	X	X	X

Self-perceived functional status	Sickness Impact Profile 68 (SIP 68; domains: mobility control, mobility range, social behaviour) [44]	X	X	X	X

**Secondary outcome measures**					

***ICF: Body functions***					

ALS disease severity	ALS Functional Rating Scale Revised (ALSFRS-R) [45]	X	X	X	X

Survival	Survival in months post diagnosis	X	X	X	X

Cardio-respiratory fitness	Submaximal exercise test with cycle ergometer [49]	X	X	X	X

Functional capacity	Stairs test [50]	X	X	X	X
	Timed Up and Go (TUG) test [51]	X	X	X	X

Muscle strength	MicroFET handheld dynamometer	X	X	X	X
	Jamar handheld dynamometer	X	X	X	X

Lung function	Spirometry: forced vital capacity (FVC) [52]	X	X	X	X
	Sniff nasal inspiratory pressure (SNIP) [53]	X	X	X	X

Fatigue	Checklist Individual Strength (CIS; domain: fatigue) [54]	X	X	X	X

Sleep disturbances	Nottingham Health Profile (NHP; sleep dimension) [55]	X	X	X	X

Pain	Visual analogue scale, pain (VAS-pain)	X	X	X	X

Co-morbidity	Cumulative Illness Rating Scale (CIRS) [56]	X			

	Blood pressure	X	X	X	X
	Height / Weight	X	X	X	X
	Skinfold	X	X	X	X

***ICF: Personal Factors***					

Illness cognitions	Ziekte Cognitie Lijst (ZCL) [57]	X	X	X	X

Coping	Coping Inventory for Stressful Situations (CISS-21) [58]	X	X	X	X

General self-efficacy	General Self-Efficacy Scale (ALCOS-16) [59]	X			

Personality	Eysenck Personality Questionnaire (EPQ-RSS) [60]	X			

Mood	Hospital Anxiety and Depression Scale (HADS) [32]	X	X	X	X
	Profile of Mood States (POMS) [61]	X	X	X	X

Impact of events	Schokverwerkingslijst (SVL) [62]	X	X	X	X

***ICF: Environmental factors***					

Social support	Social Support List - Discrepancies (SSL-D-12) [63]	X	X	X	X

Quality of life (partner)	Short Form 36 (SF-36) [41]	X	X	X	X

Coping (partner)	Coping Inventory for Stressful Situations (CISS-21) [58]	X	X	X	X

Mood (partner)	Hospital Anxiety and Depression Scale (HADS) [32]	X	X	X	X
	Profile of Mood States (POMS) [61]	X	X	X	X

Caregiver strain (partner)	Caregiver Strain Index (CSI) [46]	X	X	X	X

***Other factors***		**T0**	**T1**	**T2**	**T3**

Demographic variables (age, gender, ethnicity, marital status, children, education, work, religion, participation in other study) of patient and partner		X			

T1; pre-treatment, T2; post-treatment, T3; short-term follow-up, T4; long-term follow-up.

Participants will be asked to complete the questionnaires at home. If they are unable to complete the questionnaires themselves, e.g. due to limited hand function, they will be able to get help from the research assistant. The ALS Functional Rating Scale Revised (ALSFRS-R) [45], Hospital Anxiety and Depression Scale (HADS) [32], and Caregiver Strain Index (CSI) [46] (partner) will be administered by a rehabilitation physician at T0 and by a research assistant at T1, T2, and T3.

#### Adverse events

An adverse event is defined as any undesirable experience or outcome. All adverse events reported spontaneously by the participants or observed by the therapists will be recorded. The adverse events will be followed until they have abated, or until a stable situation has been reached.

### Statistical Analyses

Generalized estimated equations analysis will be used to investigate differences in the effects on primary and secondary outcome measures between the two intervention groups and the usual care group and to investigate the influence of possible effect modifiers. Data will be analyzed according the intention-to-treat principle. Missing data will be imputed by carrying the last observation forward.

### Power

The sample size is based on power analysis, calculated on the basis of a change in the primary outcome during the period from the baseline to 3 months post intervention. Hardly any useful data for an effective power calculation is available. A recent Cochrane review [7] showed that only two previous trials have been published [38,47] both describing results of a strength training programme, whereas our study will use aerobic exercise oriented training. The primary outcome of both studies was the ALS-FRS. Drory [47] found a significant difference between the intervention and control groups of 6.7 points, with a mean SD of 6.7 (Effect Size (ES) 1.0). Dal Bello [38] found a difference of 2.4 with a mean SD of 2.4 (ES 0.6). The ALS-FRS is a relevant outcome measure of AET, but less so for the CBT in our trial, and is therefore only a secondary outcome measure in our study. Our primary outcome measure is ALSAQ-40, a measure of QoL which is specifically designed for people with ALS. Based on the ESs of 1.0 and 0.6 found on the ALS-FRS, we expect a difference of 0.8 SD (Alpha 0.05; Beta 0.8), resulting in a minimum required number of 26 participants in each group. Allowing for a 35% drop-out rate, we aim to include 40 persons per group, so 120 people in total.

## Discussion

The FACTS-2-ALS study will evaluate the effects of AET and CTB on improving or preserving functioning and QoL in patients with ALS, compared to usual care alone. The study is characterized by some important strengths.

First, the lack of substantial evidence regarding the role of AET and CBT in people with ALS supports the need to develop further high-quality, sufficiently powered trials. The FACTS-2-ALS study design is a multicentre, single-blinded, randomized controlled clinical trial with long-term follow-up. Patients will be followed up until 6 months after the intervention, which will not only provide information about the maintenance of the effects, but also about any long-term adverse events. We have deliberately chosen a biphasic randomization model with postponed information. This design will prevent patients being disappointed if they are randomized to the control group. Besides, if patients in the control group were fully informed, they might become motivated to seek a similar intervention. Moreover, disappointment may affect the way patients answer the questionnaires about their health and QoL, and consequently influence the study outcomes. A postponed information model can also be considered patient-friendly, as it provides all relevant information stripped of the theoretical and practical complexities of the study design. Moreover, there is no risk of damage caused by not informing the participants, who will be clearly informed at a later stage, with an explanation of the reason why this method was chosen.

Secondly, our design incorporates the recommendations made by Dal Bello [7] regarding a protocol design, and include: (1) description of the population in terms of specific diagnostic criteria, range of disease severity, and severity of impairments and function to allow readers of the reports to assess the generalizability of the results to their patients or patient groups; (2) effective allocation concealment; (3) blinding of the outcome assessor; (4) a clear description of the exercise intervention, including the mode of exercise, intensity, progression, frequency, duration of each exercise session, duration of the entire programme, muscle groups exercised, supervision of exercise protocol, and compliance assessment; (5) level of activity at baseline; (6) monitoring of adverse events and reporting of motivations for dropout; and (7) standardized outcome measures, i.e. using well-validated outcome measures that are able to assess positive and negative effects of exercise, including measures of muscle function or aerobic capacity, functioning, fatigue, and QoL.

Thirdly, although CBT has proved to be effective in patients with chronic diseases, there are no studies confirming the effects of this approach in patients with ALS. This will be the first study to evaluate the effects of CBT and AET on functioning and QoL in patients with ALS. The treatment protocol was developed by psychologists who have experience with patients with chronic diseases in rehabilitation practice, and was customized to the specific problems in ALS.

However, the study also has some limitations. First, we expect that the inclusion of patients may be difficult, as the disease is very debilitating. Should the intended sample size prove difficult to reach, we will invite more centres to participate in the study, in order to include more patients. Secondly, some of the instruments we will use have not been tested on the population of patients with ALS. In view of the lack of evidence, we will use instruments that have been tested on patient groups with other chronic diseases. Finally, although the 6-month follow-up is a strong point of this study, the relatively long follow-up could cause a high drop-out rate, in view of the devastating nature of the disease. During the study, motivations for drop-out will therefore be closely monitored.

In conclusion, the FACTS-2-ALS study may provide further evidence that may be useful for the multidisciplinary care management of persons with ALS. Results might lead to adaptations of ALS treatment protocols, in order to improve or preserve QoL and functioning in patients with ALS.

## List of abbreviations

ALS: amyotrophic lateral sclerosis; AET: aerobic exercise therapy; CBT: cognitive behavioural therapy; QoL: quality of life; RCT: randomized controlled trial; FACTS-2-ALS: acronym for Fitness And Cognitive behavioural TherapieS/for Fatigue and ACTivitieS in ALS; PPS: Postpoliomyelitis Syndrome; FSHD: facioscapulohumeral dystrophy; FALS: familial ALS; SALS: Sporadic ALS; NMD: neuromuscular diseases; MD: myotonic dystrophy; RPE: rated perceived exertion; HRR: heart rate reserve; ICF: International Classification of Functioning, Disability and Health; VO2max: maximal oxygen consumption; AMC: Academic Medical Centre Amsterdam; UMCU: University Medical Centre Utrecht; UMCN: University Medical Centre Nijmegen.

## Authors' contributions

AvG is the primary investigator and responsible for data collection, analysis and interpretation; she also wrote the manuscript. IvdP, MP, CS, HG, EK, HvdL, RvV, MvdW, LvdB and EL have designed and are supervising the study. All authors have read and approved the manuscript.

## Competing interests

The authors declare that they have no competing interests.

## Pre-publication history

The pre-publication history for this paper can be accessed here:

http://www.biomedcentral.com/1471-2377/11/70/prepub
